# Formation of Surface Corrosion-Resistant Nanocrystalline Structures on Steel

**DOI:** 10.1186/s11671-016-1266-3

**Published:** 2016-02-01

**Authors:** Hryhoriy Nykyforchyn, Volodymyr Kyryliv, Olha Maksymiv, Zvenomyra Slobodyan, Oleksandr Tsyrulnyk

**Affiliations:** Karpenko Physico-Mechanical Institute of NAS of Ukraine, 5 Naukova Str., 79060 Lviv, Ukraina

**Keywords:** Nanocrystalline structure, Surface layer, Mechanical pulse treatment, Surface alloying, Middle carbon steel, Corrosion resistance

## Abstract

Engineering materials with nanocrystalline structure could be exploited under simultaneous action of mechanical loading and corrosion environments; therefore, their corrosion resistance is important. Surface nanocrystalline structure was generated on middle carbon steels by severe plastic deformation using the method of mechanical pulse friction treatment. This treatment additionally includes high temperature phase transformation and alloying. Using a complex of the corrosive, electrochemical and physical investigations, it was established that nanocrystalline structures can be characterized by lower or increased corrosion resistance in comparison with the reference material. It is caused by the action of two confronting factors: arising energy level and anticorrosive alloying of the surface layer.

## Background

One of the main tasks of the nanostructured material science is achieving and investigating the properties of ultradispersed ensembles of particles with the size up to 100 nm. The most widely used method of creating the nanostructured engineering materials is severe plastic deformation which enables obtaining bulk and surface nanocrystalline structures (NCS) [1–5]. The surface NCS could be formed also by the so-called mechanical pulse treatment (MPT) of the metal, which is based on high-speed friction between the treated component and a special metal tool [6, 7]. The special technological medium is supplied into the friction zone to play two roles: (a) alloying the surface layer with chemical elements present in the medium due to its thermal destruction within the contact zone and intensive mass transfer and (b) the structural-phase transformations in the material during its rapid cooling.

The physics of MPT consists in the metal surface layers above the phase transformation temperatures (900–1200 °C) during high-speed friction, simultaneous thermo-plastic deformation, and subsequent rapid cooling with a speed of 10^3^–10^4^ K/s due to heat transfer from surface layers into the coolant, strengthening tool and treated component. The NCS mainly of martensitic state with a grain size of 20–40 nm are formed by MPT in the surface layers. Microhardness achieves 6–12 GPa; therefore, such surface is defined by high wear and fatigue resistance.

It should be noted that MPT has some advantages over the other methods of severe plastic deformation: besides achieving NCS, the structural-phase transformation and saturation of the surface layer with alloying elements are realized under this treatment [6–8]. Hence, it gives the additional possibilities for a goal-aimed influence on the physico-mechanical properties of the nanostructured surface layer.

It is well known [1, 9] that dispersion of the structure increases general activity of the metal and plastic deformation activates electrochemical interaction between metal surface and corrosion environment. That is why using methods based on severe plastic deformation for obtaining NCS could be a complicated operation in corrosion environments because of a possible decrease of corrosion resistance of the metal. From the other hand, surface alloying of steel during MPT enables an improvement of not only mechanical [10, 11] but also corrosion properties.

In this connection, the aim of this work was to study corrosion properties of the NCS surface formed by MPT. The electrochemical methods were taken into account, and surface wetting was studied from the point of view of adsorption capacity of a corrosion environment.

## Methods

The studied materials were as follows: cylindrical bars 20 mm in diameter from steels 35 (0.35 %C) and 45 (0.45 %C) after annealing; corrosion environment—3 % aqueous solution of NaCl. Bars 120 mm long were hardened on the lathe equipped with a special device [12]. The tool rotating with a velocity of 50–70 ms^−1^ was pressed to the treated component rotating at 0.03–0.18 ms^−1^. This tool moved along a component axis with a velocity of 1.2 mm/rev using the mechanism of longitudinal feed of the lathe. The specific pressure on the treated component in the friction contact zone, created by the mechanism of longitudinal feed of the lathe, reached 1.0–1.6 GPa. The material of a strengthening tool is titanium alloy VT6 (Ti-6Al-4V), which secured higher temperature in the friction contact zone and, therefore, more intensive alloying of the surface layer compared with a steel tool. The details of the MPT process are described in papers [6–8]. Special coolants were used for surface alloying: for carbonization—mineral oil with low molecular polyethylene additives [13], for nitriding—10 % aqueous solution of aminil [14], for silicon saturation—polymethylsiloxane liquid PMS-100, and for nickel and boron saturation—PMS-100 with additives of their powders in a concentration of 15 and 18 % accordingly [15].

The cylinders of 20 mm in height were cut from the bars for electrochemical and corrosion resistance test. End faces were isolated by a special corrosion-resistant lubricant. The opened surfaces of the specimens were degreased, and the specimens were kept in the exsiccator. The corrosion resistance was studied by the gravimetric analysis [16], calculating corrosion rate *K* (μg/m^2^ · year), depth index *P* (mm/year), and a factor of corrosion resistance increase *Z*_MPT_ (*Z*_MPT_ = 100 % (*K*_ref_ − *K*_MPT_)/*K*_ref_, where *K*_ref_ and *K*_MPT_ are corrosion rates of the surface before and after MPT, correspondingly) due to MPT (in the case of the positive result). For this purpose, the previously weighed specimens were exposed into the corrosion environment for different times from 99 to 288 h. After that, the specimens were dried, lubricant was removed, and degreased, and after that, they were once more dried and weighed. Effect of MPT on corrosion resistance of the surface was established by comparing the corresponding values for grinded surface of roughness not more than *R*_а_ = 0.63 μkm.

The working surface of 0.44 mm^2^ was marked out on a specimen for electrochemical tests. The remaining surface was isolated by a lubricant. Polarization was done by the potentiostate IPC–Pro, and the polarization curves were built. Potential scanning rate was equal to 2 mV s^−1^. The saturated chloride silver electrode was used as a reference one. The corrosion current density *i*_cor_ was obtained by extrapolating anodic and cathodic Tafel lines located adjacent to the linearized current regions of the polarization curves.

Limiting wetting angles *θ* were measured by a drop method [17], which allowed the estimation of the influence of MPT on adsorption capacity of liquid environments as a precondition of their corrosion activity. As for the environment, not only water but also the aqueous solutions of alcohols of homologous series of methanol and monocarboxylic acids with different number of groups in the chain (–СН_2_–) were used. It is explained by the fact that such type of environments are potentially operating and as it will be shown in this work: the effect of MPT on *θ* is opposite for water and for the organic compounds. Since it is impossible to measure a limiting wetting angle for steel surfaces by alcohols and acids because of their complete spreading on the surface, their aqueous solution (10^−2^ М) was studied. This is acceptable, taking into account that the surface-active substances concentrate in the surface layers of a drop and contact directly with the metal.

## Results and Discussion

The X-ray analysis gives a possibility to establish that MPT generates the ferrite-austenite NCS on the steel surface independently of coolant type. As the example, the data for the steel 45 after MPT with coolant for carbonization are presented in Table 1.

**Table 1:** The data X-ray analysis in Fe*α*-radiation with *λ* = 1936 Å of the specimen surface of the steel 45

Crystallite size, nm	Dislocation density, *ρ* · 10^12^ сm^−2^	*α*-Fe, %	*γ*-Fe, %
19	0.86	78	22

**Fig. 1:**
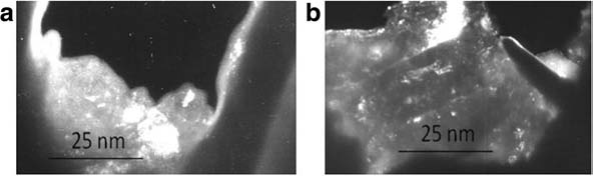
The electron microscope images of the steel 45 structure. Structures have made at a depth of **a** 10 μm and **b** 15 μm from the surface after MPT in the coolant for carbonization

**Fig. 2:**
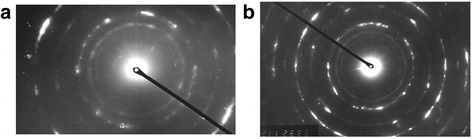
The diffraction patterns of the steel 45 structures. Diffraction patterns have made at a depth of **a** 10 μm and **b** 15 μm from the surface after MPT in the coolant for carbonization

The electron microscopy images of the steel 45 microstructure at different depths after MPT are presented in Fig. 1. The diffraction patterns at the same depth are presented in Fig. 2. Such diffraction patterns are typical of NCS. It is obvious that structure dispersion is high and it increases approaching the surface. It is well known [1] that NCS obtained by severe plastic deformation has such peculiarities as the presence of nonequilibrium grain boundaries and a great number of triple junctions, which are a source of great internal stresses. Diffusion contrast of boundaries and bended shapes of grain boundaries in the crystallites, presented in Fig. 1, confirm such conclusion. It is worthy to add that nonequilibrium boundaries of NCS are characterized by high dislocation density, creating far acting fields of stresses, which are a reason of excess energy of grain boundaries [1]. Thus, one can suppose that surface activation after MPT could intensify the corrosion processes.

The results of corrosion studies of steels are presented in Table 2. Corrosion rate *K* and depth index *P* of untreated specimens (without MPT) decrease with a growing test time. It agrees with general principles of corrosion processes in the neutral environments and is explained by some protective properties of corrosion products. Note that the steel 45 with a higher content of carbon undergoes corrosion more intensively than the steel 35, thus corresponding to the general rule about the negative influence of carbon content in steels on its corrosion rate.

**Table 2:** Corrosion resistance of steels 35 and 45 in 3 % aqueous solution of NaCl

Material	Type of coolant	Test time, h	K · 10^4^ mg/m^2^ · h	*P*, mm/year	*Z*, %
Steel 35	Untreated	99	650	0.72	–
		192	630	0.70	
		288	600	0.65	
	Carbon	99	780	0.86	–
	Silicon	99	850	0.94	–
	Nitrogen	99	765	0.84	–
	Nickel	288	120	0.13	80
	Boron	288	310		48
Steel 45	Untreated	99	780	0.87	–
		192	710	0.79	
		288	700	0.77	
	Carbon	192	760	0.84	–
	Silicon	192	570	0.63	20
	Nitrogen	288	330	0.36	53

The MPT can both lower and promote a corrosion rate. It is doubtless that alloying of surface layer with carbon intensifies the metal corrosion and it is in agreement with the above-said role of carbon in the corrosion processes. Concerning the effect of MPT alloyed with silicon and nitrogen, the result was negative for the steel 35 and positive for the steel 45. It should be mentioned that the severe plastic deformation should increase the corrosion activity of the metal while alloying would have only an additional influence on this process. That is why the negative influence of MPT must not be connected with the surface layer alloying. Besides, it should be taken into consideration that MPT of metal with higher carbon content (steel 45) increases the quantity of retained austenite and this can have the influence on corrosion processes of the metal too.

The most positive effect on corrosion resistance was observed for the specimens alloyed with boron, nitrogen, and nickel. The value of *Z* is equal to 48, 53, and 80 %, respectively. These variants of the treatment could be considered as the most promising.

**Fig. 3:**
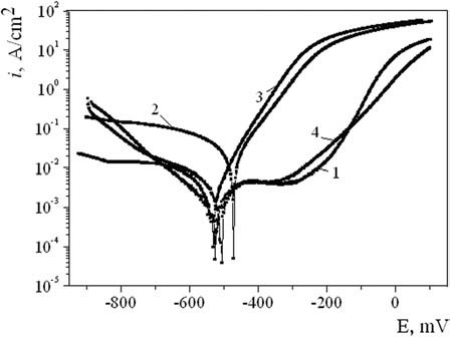
Polarization curves of the steel 45 in 3 % aqueous solution of NaCl after MPT. MPT realized with using different coolants for alloying: (*1*) untreated; (*2*) carbon; (*3*) silicon; (*4*) nitrogen

Polarization curves are presented in Fig. 3 and the results of their analysis in Table 3. Corrosion currents decrease in a raw of specimens: untreated > MPT with carbon alloying > MPT with silicon alloying > MPT with nitrogen alloying. These results correspond well with the data of gravimetric evaluation of the corrosion rate (Table 2). The exception is for the specimens alloyed with carbon, when gravimetric evaluated corrosion rate somewhat increases and corrosion current slightly decreases. In this connection, it should be noted that polarization curves are built after 15–30 min of specimen exposition in the corrosion environment, when corrosion potential *E* is stabilized, while the parameters *K* and *P* were calculated on the base of corrosion tests duration of 99–288 h. Concerning the corrosion potential, its correlation with the corrosion rate is ambiguous under the shift of *E* level by MPT to the positive direction. So, the biggest one was observed in the case of MPT with carbon alloying. But such variant of MPT can’t be considered as a positive taking into account changes of *K* and *P*. On the other hand, the corrosion potential after alloying with nitrogen has changed insignificantly, but the positive effect is huge.

**Table 3:** Electrochemical characteristics of the steel 45 in 3 % aqueous solution of NaCl after MPT with different coolants

Type of coolant	Corrosion potential, −Е, mV	Corrosion current density *i*_cor_ · 10^4^, А/сm^2^
Untreated	535	0.54
Carbon	470	0.50
Silicon	525	0.46
Nitrogen	512	0.38

**Fig. 4:**
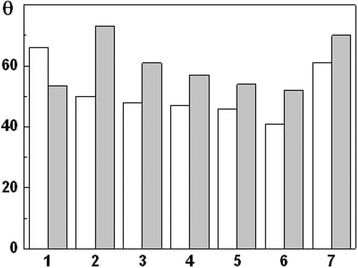
Influence of aliphatic alcohols on the limiting wetting angles *θ* for the steel 45. (*White*) Untreated; (*gray*) after MPT: (*1*) Н_2_О; (*2*) С_2_Н_5_ОН; (*3*) С_5_Н_11_ОН; (*4*) С_6_Н_13_ОН; (*5*) С_7_Н_15_ОН; (*6*) С_8_Н_17_ОН; (*7*) С_10_Н_21_ОН

**Fig. 5:**
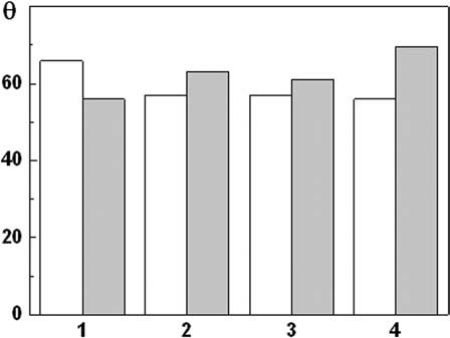
Influence of monocarboxylic acids on the limiting wetting angles *θ* for the steel 45. (*White*) Untreated; (*gray*) after MPT: (*1*) Н_2_О; (*2*) С_2_Н_5_СООН; (*3*) С_4_Н_9_СООН; (*4*) С_5_Н_11_СООН

General characteristics of aliphatic alcohols and monocarboxylic acids used for investigation of the metal surface wetting are presented in Table 4. The surface tension was evaluated by the method of the highest pressure in the air bubble (Rebinder’s method) [17]. The measurements of limiting wetting angles showed (Figs. 4 and 5) that MPT decreased *θ* by about 10 %; in other words, the surface with nanolayer hydrophilized, as a result of the interaction between surface and water is more intensive. Hence, corrosion intensification after MPT can be predicted.

**Table 4:** Physico-chemical characteristic of aliphatic alcohols and monocarboxylic acids

Compound	Molecular weight	Surface tension, σ · 10^−3^, N/m	Water solubility, g/100 g Н_2_О
Н_2_О	18	72.75	–
С_2_Н_5_ОН	46	22.03	∞
С_5_Н_11_ОН	88	25.16	2.6
С_6_Н_13_ОН	102	24.08	1.0
С_7_Н_15_ОН	116	24,48	0.09
С_8_Н_17_ОН	130	26.06	–
С_10_Н_21_ОН	158	26.72	–
С_2_Н_5_СООН	74	26.7	∞
С_4_Н_9_СООН	102	26.35	3.7
С_5_Н_11_СООН	116	28.05	0.89

The symbol ∞ means unlimited water solubility of this compound

On the other hand, the surface tensions of the investigated aliphatic alcohols and monocarboxylic acids are much lower than *θ* of the water. In this case, the increase of the length of carbon backbone chain in a molecule decreases the limiting wetting angles. As for influence of MPT, the opposite effect was revealed relatively to the experiment with the water. The limiting wetting angles of the surface hardened by MPT are higher than untreated specimens. It means that the nanocrystalline layers, formed by MPT, promote hydrophobization of the surface in the solutions of organic matters and, potentially, weaken the interaction between the surface and corrosion environment.

## Conclusions

1. Mechanical pulse friction treatment which combines severe plastic deformation, surface alloying, and structural-phase transformations gives the nanocrystalline structure of the surface for middle carbon steels (~20 nm). X-ray and electron microscope analyses established diffusion contrast and bended shape of the grain boundaries in the crystallites, illustrating the nonequilibrium grain boundaries, which are a source of significant internal stresses. Since such structures are characterized by high dislocation density, they create far acting fields of stresses, which are a reason of excess energy of grain boundaries. So, it is expected that surface activation after MPT will intensify the corrosion processes.
2. Surface mechanical pulse treatment can both increase or decrease the corrosion rate. It is connected with an action at least of two confronting factors: severe plastic deformation should increase corrosion activity of the metal while anticorrosion alloying decrease it. The alloying with nitrogen, nickel, and boron is the most effective.
3. Calculated corrosion rates obtained from the results of electrochemical investigations generally agree with metal corrosion evaluations done by the gravimetric analysis. However, corrosion potential is not a representing parameter, since it moves to positive values independently of the character of the treatment influence on the corrosion rate.
4. The measurements of limiting wetting angles by water showed that hardening treatment hydrophilizes the surface and, because of that, its interaction with water intensifies. Hence, the corrosion intensification can be predicted. However, the opposite effect was obtained in the case of using aliphatic alcohols and monocarboxylic acids. The treatment promotes the hydrophobization of the surface in the solutions of organic matters and, potentially, weakens the interaction between the surface and the environment. It is important to take this into account in case the working aqueous environments contain organic impurities.

## References

[1] Valiev RZ, Islamgaliev RK, Aleksandrov IV (2000). Bulk nanostructured materials from severe plastic deformation. Progress Mat Sci.

[2] Lu K, Lu J (1999). Surface nanocrystallization (SNC) of metallic materials-presentation of the concept behind a new approach. Mater Sci Technol.

[3] Yurkova A, Belots’ky A, Byakova A, Podrezov Yu, Danylenko M. Nanocrystallization in iron alloys induced by friction treatment and nitrogen diffusion. *In: Metallic materials with high structural efficiency*. Nitherlands: Kluver Academic Publishers; 2004. p. 113-8.

[4] Wang ZB, Tao NR, Li S (2003). Effect of surface nanocrystallization on friction and wear properties in low carbon steel. Mater Sci Eng.

[5] Wu X, Hong Y, Lu J, Lu K (2002). Fabrication and nanostructured surface layers of Al alloy by surface vibrational mechanical attrition. Mat Res Soc Symp Proc.

[6] Nykyforchyn HM, Kyryliv VI, Slobodjan DV (1998). Structural steels surface modification by mechanical pulse treatment for corrosion protection and wear resistance. Surf Coat Technol.

[7] Nykyforchyn H, Kyryliv V, Maksymiv O. Physical and mechanical properties of surface nanocrystalline structures, generated by severe thermal-plastic deformation. *In: Fesenko O,Yatsenko L, editors*. Nanocomposites, Nanophotonics, Nanobiotechnology, and Applications, Springer Proceedings on Physics 156. Switzerland: Springer International Publishing; 2014. p. 31–41

[8] Nykyforchyn H, Lunarska E, Kyryliv V, Maksymiv O (2015). Influence of hydrogen on the mechanical properties of steels with the surface nanostructure. Nanoplasmonics, Nano-Optics, Nanocomposites, and Surface Studies Springer Proceedings in Physics.

[9] Buckleu DH (1981). Surface effects in adhesion, friction, wear, and lubrication.

[10] Kuzydlowski KJ (2006). Physical, chemical, and mechanical properties of nanostructured materials. Mater Sci.

[11] Nykyforchyn H, Kyryliv V, Maksymiv O (2015). Effect of nanostructurisation for structural steels on their wear hydrogen embittlement resistance. Solid State Phenom.

[12] Kalichak TN, Kyryliv VI, Fenchin SV (1989). Mechanopulsed hardening of long components of the hydraulic cylinder rod type. Sov Mater Sci.

[13] Kyryliv VI (1999). Surface saturation of steel with carbon during mechanical-pulse treatment. Mater Sci.

[14] Kyryliv VI (2013). Nitriding of steels in the course of their mechanical pulsed treatment. Mater Sci.

[15] Kyryliv VI, Koval’ YM (2001). Surface alloying of steels from special process media. Mater Sci.

[16] Kyryliv VI, Slobodyan ZV, Sydor PY, Linyns’ka OD (2003). Effect of surface alloying in the course of mechanical-pulse treatment on the corrosion resistance of steel. Mater Sci.

[17] Slobodyan ZV, Mahlatyk LA, Kupovych RB (2007). Influence of the plastic deformation of steels on the process of wetting of their surfaces with KORSOL-type inhibitors. Mater Sci.

